# Retraction: Population dynamics in pre-Inca human groups from the Osmore Valley, the Azapa Valley and the coast of the South Central Andes

**DOI:** 10.1371/journal.pone.0348927

**Published:** 2026-05-11

**Authors:** The PLOS One Editors

After this article [1] was published, concerns were raised about reuse of data without the relevant permissions.

Specifically, several datasets used in this article [1] originate from a 1997 PhD dissertation [2] (reference 23 in [1]), a dental morphology study on the Peruvian Late Intermediate sites of Chiribaya, Yaral, and San Geronimo, and Chilean sites from the Azapa Valley, which was combined with a separate dataset gathered for [1] as described in the Materials and methods section of [1]. These datasets are described in Table 1 in [1] as groups 1–5 and 8–12 (incorrectly attributed to “Sutter 1987” in the Data Origin column of Table 1 in [1]) and are presented in S2-S7 and S9-S13 Tables in [1]. This data has been included in [1] without the authorization of the dissertation’s author and data owner.

The corresponding author stated that as [2] was publicly available, they believed that the usage of this data was permissible, provided it was correctly cited. They also stated that S2–S7 and S9–S13 Tables in [1] do not reproduce in full the data from [2], and only report the data relating to the dental traits used in [1] for comparative purposes. They stated that the traits Winging maxillary central incisor, Distosagittal Ridge maxillary premolars, Odontome maxillary premolars, Tome’s Root mandibular first premolar, and Odontome mandibular premolars were not included in S2–S7 and S9–S13 Tables in [1]. The *PLOS One* Editors consider that the data from [2] contribute significantly to the results and conclusions of [1].

In light of the above concerns about the reuse of data in [1] without proper authorization, the *PLOS One* Editors retract this article.

ACoppa did not agree with the retraction. FC, CA, EdlVM, EGMT, ML, and ACucina either did not respond directly or could not be reached.

Table 1, S2-S7 Tables, and S9-S13 Tables report material from [2], published in 1997 by Richard Carlton Sutter, which are not offered under a CC BY license and are therefore excluded from this article’s [1] license.

## References

[1] Coppa A, Candilio F, Arganini C, de la Vega Machicao E, Moreno Terrazas EG, Lucci M, et al. Retracted: Population dynamics in pre-Inca human groups from the Osmore Valley, the Azapa Valley and the coast of the South Central Andes. PLoS One. 2020;15(12):e0229370. doi: 10.1371/journal.pone.0229370 33326416 PMC7743979

[2] Sutter RC. Dental variation and biocultural affinities among prehistoric populations from the coastal valleys of Moquegua, Perú, and Azapa, Chile. University of Missouri-Columbia; 1997.

